# Supplementary material for: A novel role for phospholamban in the thalamic reticular nucleus
Source: Sci Rep. 2024 Mar 16;14:6376. doi: 10.1038/s41598-024-56447-x (PMC10944534; doi:10.1038/s41598-024-56447-x)
Supplement: Supplementary file 1 — Supplementary Information. [file 41598_2024_56447_MOESM1_ESM.pdf]

SUPPLEMENTARY MATERIAL

Title: A Novel Role for Phospholamban in the Thalamic Reticular Nucleus
Abbreviated Title: A Novel Role for PLN in the TRN

Authors: Benjamin Klocke[1+], Aikaterini Britzolaki[1+], Joseph Saurine[1], Hayden Ott[1], Kylie Krone[1], Kiara Bahamonde[1], Connor Thelen[1], Christos Tzimas[2], Despina Sanoudou[2,3], Evangelia G. Kranias[2,4], Pothitos M. Pitychoutis[1*]

Affiliations:
[1] Department of Biology, University of Dayton, Dayton, Ohio 45469, USA.
[2] Molecular Biology Department, Biomedical Research Foundation of the Academy of Athens, Athens, 11527, Greece
[3] 4th Department of Internal Medicine, Clinical Genomics and Pharmacogenomics Unit, Medical School, "Attikon" Hospital, National and Kapodistrian University of Athens, 11527 Athens, Greece.
[4] Department of Pharmacology and Systems Physiology, University of Cincinnati College of Medicine, Cincinnati, Ohio 45267, USA.

[+]: Equal Contribution

***: Corresponding Author**
Pothitos M. Pitychoutis, Ph.D.
Associate Professor in Neuroscience & Neuroscience Minor Program Coordinator
Department of Biology, University of Dayton
300 College Park, Dayton, OH 45469-2320
Office: Science Center 223B; Office Phone: (937)-229-2287
Email: ppitychoutis1@udayton.edu

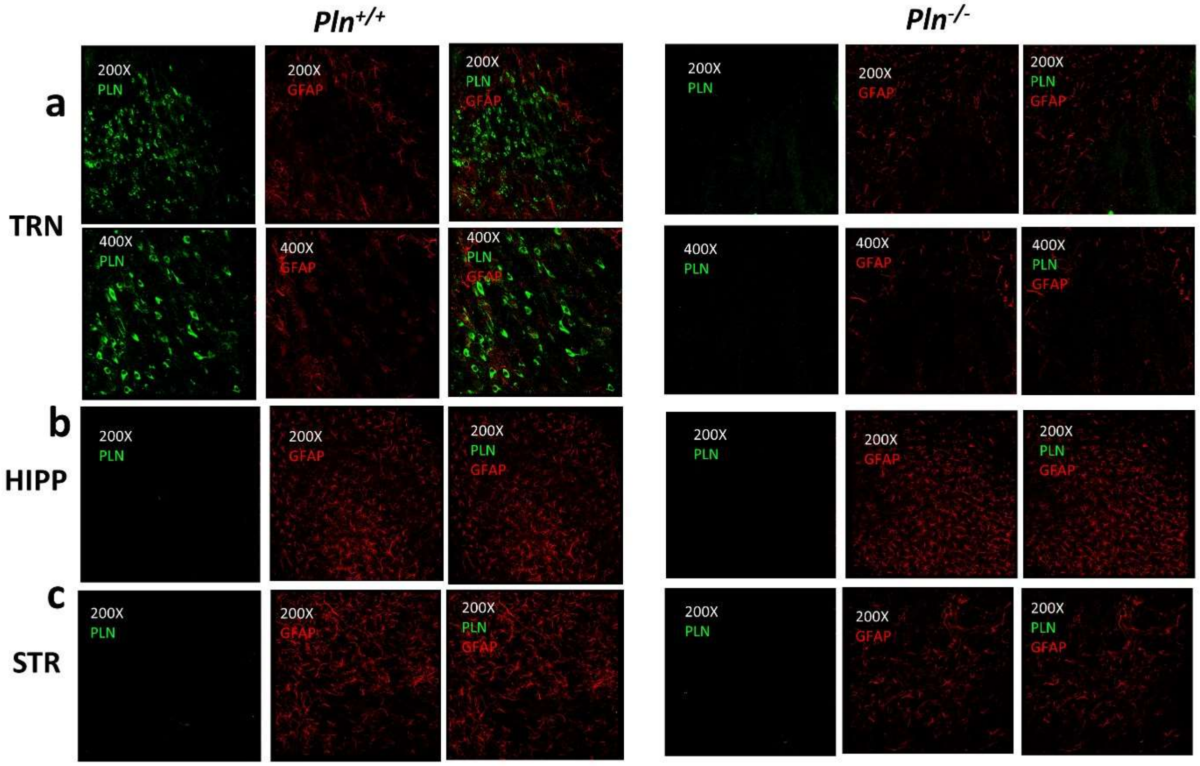

Supplementary Figure S1: PLN immunoreactivity was not detected in the astrocytes of the mouse brain: PLN immunoreactivity was not detected in astrocytes in a) the thalamic reticular nucleus (TRN); b) hippocampus (HIPP); and c) the striatum in our experimental setup. PLN and GFAP protein expression was assessed using fluorescent IHC in coronal free-floating brain sections from adult *Pln*$^{+/+}$ and *Pln*$^{-/-}$ mice stained with mouse monoclonal PLN (1:100), and rabbit polyclonal GFAP (1:500) primary antibodies. Representative images were obtained at 200X and 400X magnifications using an Olympus Fluoview 3000 confocal microscope (Olympus, MA, USA).

SUPPLEMENTARY TABLE S1

Supplementary Table S1: Statistically significant results of statistical analyses for behavioral experiments.

Paradigm or assay	Parameter measured	N/ genotype/sex	Statistical test	Comparison	Statistics	Deg. freed.	P value	Fig.
**OFT**	Distance travelled	17-24	Three-way RM ANOVA	Main effect of genotype	F=11.201	1, 75	<0.001	**4a**
				Main effect of time	F=120.229	4.999, 347.947	<0.001	
	Time in center	17-24	Kruskal-Wallis	*Pln*$^{+/+}$ vs. *Pln*$^{-/-}$			0.047	**4b**
**Y-maze**	% Spontaneous Alternation score	17-24	Two-way ANOVA	Main effect of genotype	F=7.887	1, 30	0.009	**4c**
**OLM**	Interaction time with objects	6-12	2-way ANOVA	Main effect of object	F=27.19	1,72	<0.001	**4d**
**ORM**	Interaction time with objects	6-12	2-way ANOVA	Main effect of object	F=11.14	1,72	0.001	**4e**
				Object x genotype interaction	F=4.411	1,72	0.039	
			Bonferroni's multiple comparisons	*Pln*$^{+/+}$: Familiar vs Novel			0.0029	
**3-chamber social interaction test: Social I vs empty**	Interaction time	5	Two-way ANOVA	Main effect of stimulus	F=24.18	1, 36	<0.001	**4f**
**3-chamber social interaction test: Social I vs Social II**	Interaction time	5	Two-way ANOVA	Main effect of stimulus	F=19.37	1, 36	<0.001	**4g**
**OFT**	Distance travelled (5min bins)	11-16	Three-way RM ANOVA	Main effect of time	F=40.085	3.717, 193.281	<0.001	**5b**
				Time x genotype interaction	F=6.685	3.717, 193.281	<0.001	
	Distance travelled (15min)	11-16	One-way ANOVA	Main effect of genotype	F=7.403	1, 52	0.009	**5c**
**EEG Sleep Analysis**	Total: REM duration	3-5	Two-way ANOVA	Sex x genotype interaction	F=9.248	1, 16	0.01	**6c**
			Bonferroni's multiple comparisons	Control ♀ vs cKO ♀			0.048	

	Dark period: Wakefulness duration	3-5	Two-way ANOVA	Main effect of genotype	F=6.171	1,16	0.029	**6d**
	Dark period: Wakefulness bout length	3-5	Two-way ANOVA	Main effect of genotype	F = 8.068	1, 12	0.0149	**6d**
	Dark period: NREM duration	3-5	Two-way ANOVA	Main effect of genotype	F=5.291	1, 16	0.04	**6e**
	Dark Period:No. of NREM bouts	3-5	Two-way ANOVA	Main effect of genotype	F = 4.805	1, 12	0.0488	**6e**
	Dark period: REM duration	3-5	Two-way ANOVA	Main effect of genotype	F=3.481	1, 16	0.024	**6f**
				Sex x genotype interaction	F=17.934	1, 16	0.001	
			Bonferroni's multiple comparisons	Control ♀ vs cKO ♀			0.002	
	Dark Period: No of REM bouts	3-5	Two-way ANOVA	Sex x genotype interaction	F = 9.937	1,12	0.0083	**6f**
			Bonferroni's multiple comparisons	Control ♀ vs cKO ♀			0.0494	
				control ♀ vs control ♂			0.0294	
	Dark Period: REM bout length	3-5	Two-way ANOVA	Main effect of genotype	F = 8.458	1, 12	0.0131	**6f**
				Sex x genotype interaction	F = 8.887	1, 12	0.0115	
			Bonferroni's multiple comparisons	Control ♀ vs cKO ♀			0.0089	
	Dark Period: Wake-NREM transitions	3-5	Two-way ANOVA	Main effect of genotype	F=5.430	1, 12	0.0381	**6g**
	Dark Period: NREM-Wake transitions	3-5	Two-way ANOVA	Main effect of genotype	F = 4.206	1, 12	0.0628	**6h**
	Dark Period: No. of NREM arousals	3-5	Two-way ANOVA	Main effect of genotype	F = 4.677	1, 12	0.0515	**6i**

**5-CSRTT: FR1**	FR1 acquisition	5-9	Mantel-Cox Log-rank test	cKO ♀ vs ♂ cKO	x^2=5.753	3	0.016	**7b**
				cKO ♀ vs control ♂	x^2=5.560	3	0.018	
				cKO ♀ vs control ♀	x^2=3.171	3	0.075 (trend)	
**5CSRTT: Titration**	Premature response rate	5-9	Two-way ANOVA	Main effect of genotype	F=4.052	1, 24	0.055 (trend)	**7j**
	Premature responses (total)	5-9	Two-way ANOVA	Main effect of genotype	F=4.273	1, 24	0.05	**7k**

	Total		Light Period		Dark Period	
	**Control**	**cKO**	**Control**	**cKO**	**Control**	**cKO**
Wakefulness (% of total power)						
**Delta**	52.389±5.414	45.146±2.692	55.906±5.587	50.207±2.327	50.412±5.286	42.171±2.986
**Theta**	30.923±3.454	35.568±2.06	29.189±3.318	33.236±2.039	31.876±3.478	36.922±2.11
**Alpha**	9.513±1.389	10.487±0.844	7.912±1.432	8.664±0.607	10.335±1.396	11.557±1.019
**Beta**	7.148±1.575	8.751±1.236	6.978±1.737	7.84±0.84	7.341±1.519	9.306±1.493
**Gamma**	0.027±0.015	0.048±0.021	0.015±0.007	0.053±0.019	0.035±0.022	0.044±0.023
NREM Sleep (% of total power)						
**Delta**	55.219±5.864	44.586±2.516	55.432±6.22	44.611±2.759	53.239±4.305	44.694±2.348
**Theta**	25.059±2.519	29.456±1.117	24.322±2.54	28.904±1.238	27.169±1.884	30.498±1.047
**Alpha**	10.168±1.933	12.901±0.976	10.443±2.062	13.486±1.019	10.055±1.587	11.931±0.898
**Beta**	9.55±1.89	13.054±1.788	9.798±2.013	12.994±1.605	9.535±1.531	12.873±2.125
**Gamma**	0.004±0.001	0.004±0.001	0.004±0.001	0.004±0.002	0.003±0.001	0.004±0.001
REM Sleep (% of total power)						
**Delta**	38.709±5.149	29.142±2.149	39.075±5.452	28.625±2.009	36.628±3.69	30.155±2.374
**Theta**	37.571±2.854	42.951±1.957	36.952±2.942	42.956±1.84	39.799±2.317	42.82±2.401
**Alpha**	12.686±1.72	14.129±0.755	12.707±1.765	14.443±0.639	12.977±1.556	13.611±0.951
**Beta**	11.029±1.495	13.772±2.301	11.26±1.515	13.97±2.135	10.593±1.433	13.409±2.754
**Gamma**	0.004±0.001	0.005±0.002	0.005±0.001	0.005±0.002	0.003±0.001	0.005±0.002

Supplementary Table S2: Conditional deletion of *Pln* in the TRN does not affect sleep spectral profile: No differences detected in EEG spectral profile in delta, theta, beta, alpha, or gamma frequency bands during wakefulness, NREM, or REM sleep when assessing the total, light, or dark period. Data is expressed as power (μV^2) of delta (δ; 0.5-4Hz), theta (θ; 4-8Hz), alpha (α; 8-13Hz), beta (β; 13-30Hz), gamma (γ; 30-40Hz), frequency bands, normalized as a percentage of total power across all frequency bands.

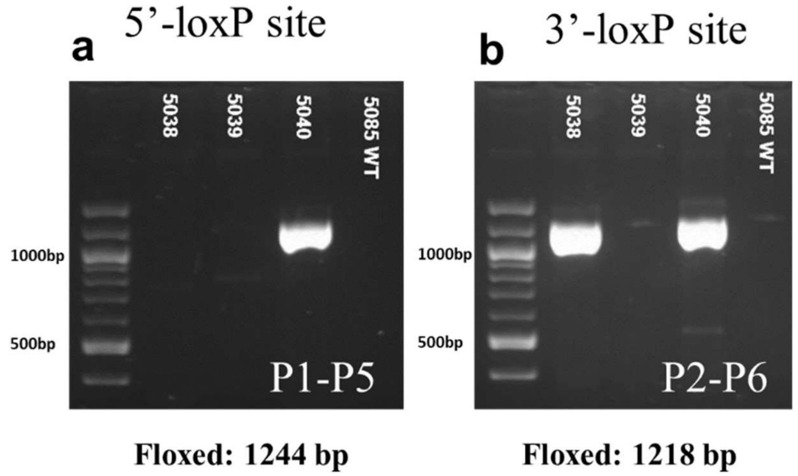

Supplementary Figure S2: Original PCR gel images for Figure 1b. During the construction of the floxed *Pln* mouse line, mice were genotyped for the **a)** 5’-loxP site and the **b)** 3’-loxP site flanking the *Pln* locus, using the following primers: P1: AGAGGCTCTGTGTCTCCAGTAAACAC, P2: CCAAGAGGCTGGATCTATAACTTC, P5: CGTTGCTTCCCCCAAGCTTATAAC, and P6: AGACTGGAGCTATAAAGTGACCTTGC. Mouse #5040 was chosen as the mosaic floxed mouse due to the presence of both the 5’-loxP and the 3’-loxP sites.

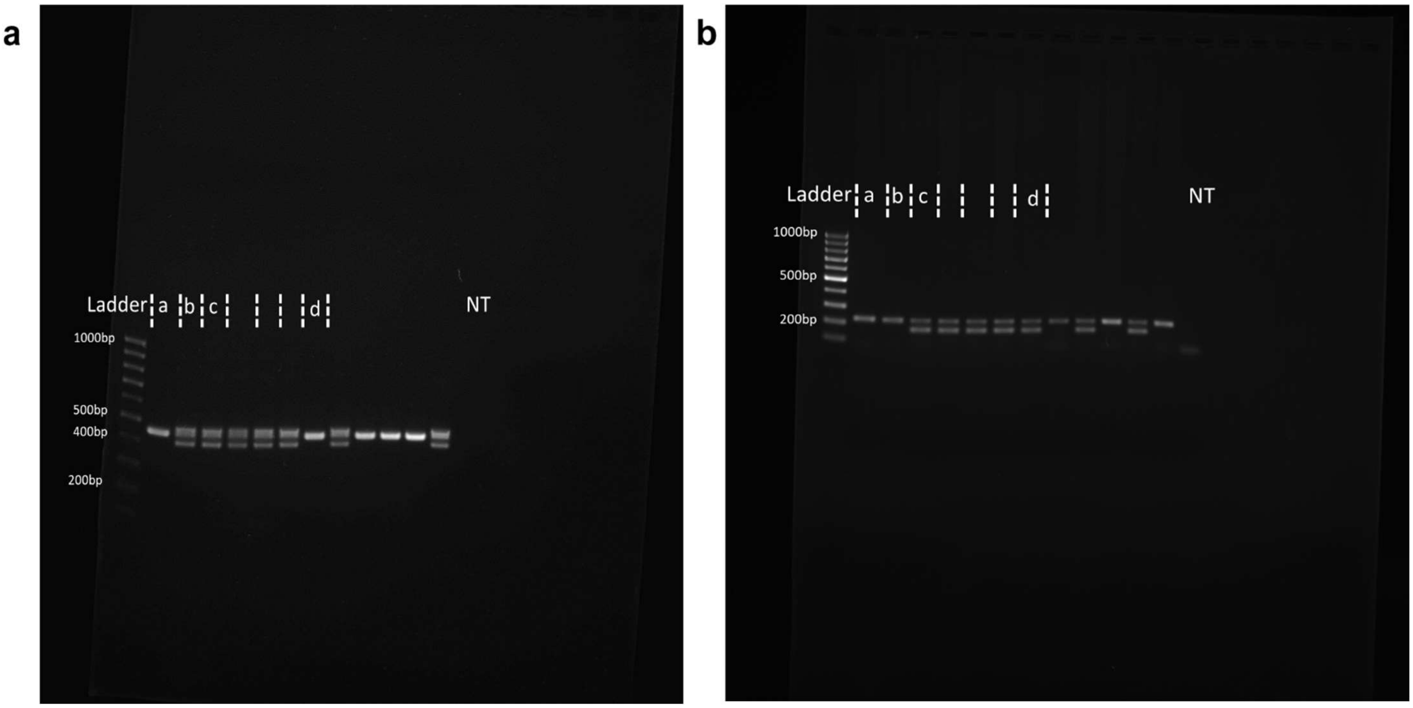

Supplementary Figure S3: Original PCR gel images for Figure 1e. Representative **a)** floxed *Pln* and **b)** *Gad2-Cre* genotyping results from a litter of cKO mice. As expected for floxed *Pln* genotyping, WT allele bands are 414bp, and floxed *Pln* alleles are 453bp. For all experiments, both control and cKO mice were $Pln^{lox/lox}$. As expected for *Gad2-Cre* genotyping, WT allele bands are 225bp, and Cre alleles are 176bp. For all experiments, control mice were $Gad2\text{-}Cre^{0/0}$, while cKO mice were $Gad2\text{-}Cre^{0/+}$. Genotyping results from corresponding samples displayed in Fig. 1e are labeled as a: $Pln^{lox/lox}$;$Gad2\text{-}Cre^{0/0}$ (*i.e.,* Control); b: $Pln^{lox/WT}$;$Gad2\text{-}Cre^{0/0}$; c: $Pln^{lox/WT}$;$Gad2\text{-}Cre^{0/+}$, and d: $Pln^{lox/lox}$;$Gad2\text{-}Cre^{0/+}$ (i.e., cKO). NT: non-template control; ddH_2O).

a

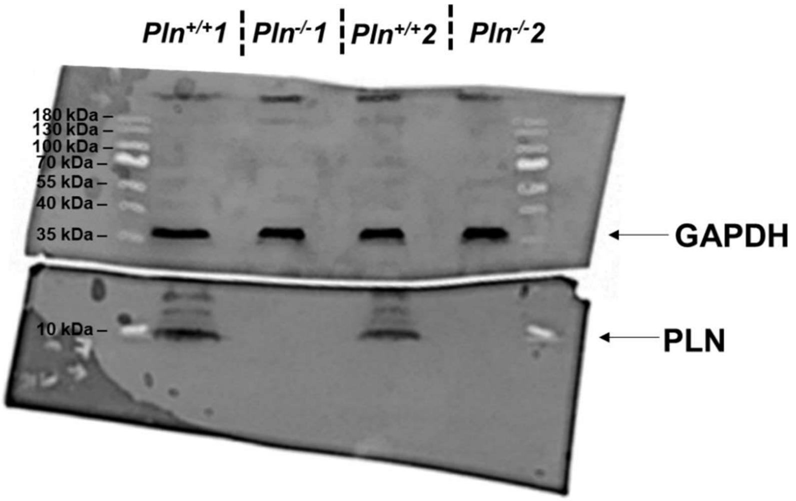

b

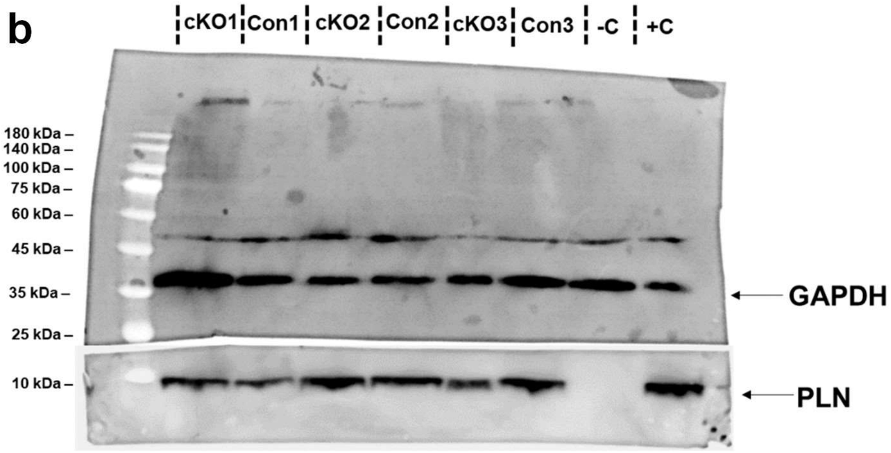

Supplementary Figure S4: Original Western blot image for Figures 1f/g. a) To confirm the validity of the anti-PLN antibody (#MA3-922, Invitrogen) used for immunohistochemical assessments, PLN protein expression was assessed via Western blotting in *Pln*$^{+/+}$ and *Pln*$^{-/-}$ mouse heart samples; PLN monomer was detected at the expected molecular weight in *Pln*$^{+/+}$ but not in *Pln*$^{-/-}$ mice. **b)** PLN protein is detected in the hearts of control (Con) and cKO mice. PLN protein expression was assessed via Western blotting in control and cKO mouse heart samples; *Pln*$^{+/+}$ and *Pln*$^{-/-}$ mouse heart samples served as positive and negative controls, respectively. Following protein transfer, membranes were cut horizontally, and antibody hybridization was performed in parallel with either anti-GAPDH antibody (a: #NB100-56875, Novus Biologicals, 1:200; b: #PA1-987, Invitrogen, 1:500) or anti-PLN antibody (#MA3-922, Invitrogen, 1:500).

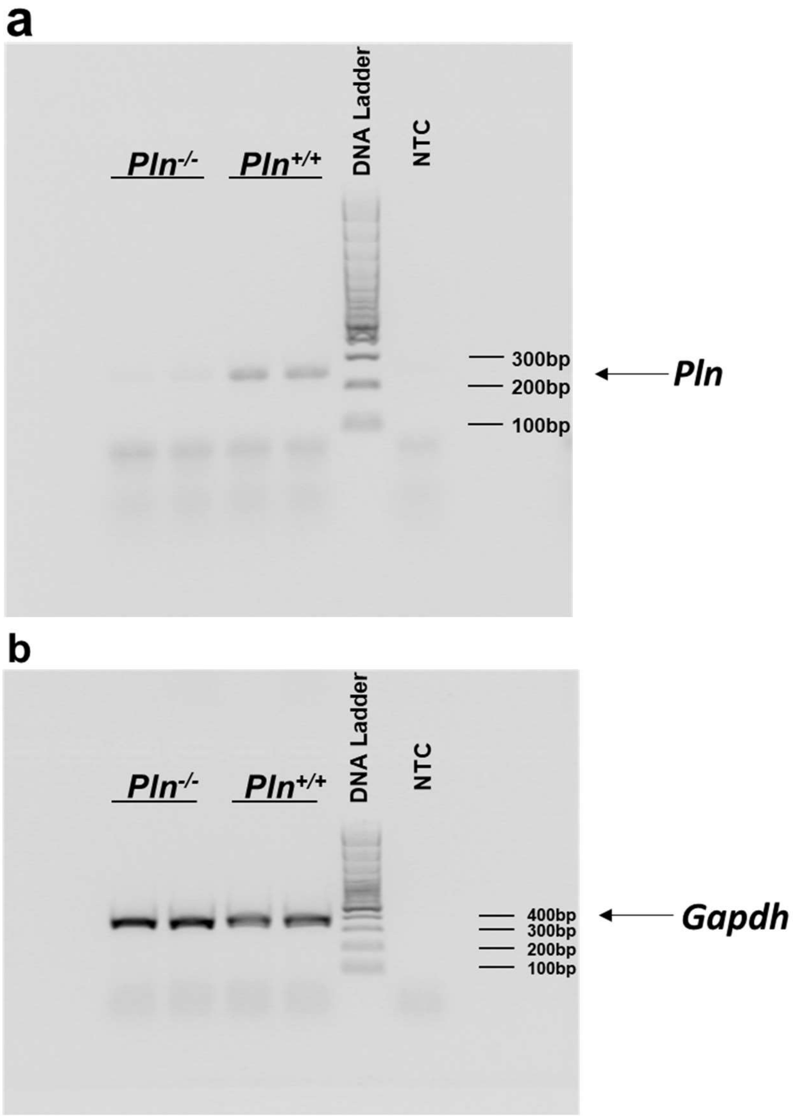

Supplementary Figure S5: Original RT-PCR gel image for Figure 2a. a) PLN mRNA expression in the whole mouse brain was assessed in *Pln*$^{+/+}$ and *Pln*$^{-/-}$ animals using RT-PCR procedures yielding a transcript variant of 235bp in *Pln*$^{+/+}$ mice; **b)** GAPDH was used as loading control. The following primers were used; PLN: Forward T1T2: 5'-CCCATAAACCTGGGAACAGA-3'; Reverse T1T2: 5'-TAGCCGAGCGAGTGAGGTAT-3'; GAPDH: Forward: 5'-ACTCCACTCACGGCAAATTC-3'; Reverse: 5'- CCTGCTTCACCACCTTCTTGAT-3'. NTC: no template control; ddH_2O.
